# The effectiveness of a nurse-led intervention with the distress thermometer for patients treated with curative intent for breast cancer: design of a randomized controlled trial

**DOI:** 10.1186/s12885-016-2565-x

**Published:** 2016-07-25

**Authors:** Floortje K. Ploos van Amstel, Judith B. Prins, Winette T. A. van der Graaf, Marlies E. W. J. Peters, Petronella B. Ottevanger

**Affiliations:** 1 Department of Medical Oncology, Radboud University Medical Center, P.O. Box 9101, 6500 Nijmegen, The Netherlands; 2 Department of Medical Psychology, Radboud University Medical Center, Nijmegen, The Netherlands; 3 Institute of Cancer Research and Royal Marsden NHS Foundation Trust, London, UK

**Keywords:** Distress, Distress thermometer, Breast cancer, Quality of life, RCT, Nurse-led intervention, Nurse, Oncology nurse, Screening, Psychosocial care

## Abstract

**Background:**

Distress in patients with cancer influences their quality of life. Worldwide, screening on distress with the Distress Thermometer (DT) in patients with cancer is recommended. However, the effects of the use of the DT on the psychosocial wellbeing of the patient are unknown. A study to assess the psychosocial consequences of the systematic use of the DT and its discussion by a nurse as compared to the usual care provided to outpatients who are treated for primary breast cancer is needed.

**Methods/design:**

The effectiveness of a nurse-led intervention with the DT will be tested in a non-blinded randomized controlled trial. Patients treated with curative intent for breast cancer will be recruited from the Radboud University Medical Center. The intervention consists of the DT together with discussion of the results with the patient by a trained oncology nurse added to the usual care. Patients will be randomly allocated (1:1) to either receive usual care or the usual care plus the intervention. Primary outcome measure is global quality of life measured with the EORTC QLQ-C30. The functional and symptom scales of the EORTC QLQ-C30 and BR23, Hospital Anxiety and Depression Scale, Impact of Event Scale, Illness Cognition Questionnaire and DT (baseline and final measurement only) will be used to measure secondary outcomes. Questionnaires are obtained in both arms at baseline, after completion of each type of cancer treatment modality and during follow up, with a three and six months’ interval during the first and second year respectively.

**Discussion:**

This study will be the first randomized controlled longitudinal study about the effectiveness of the DT as nurse led-intervention. In case of proven effectiveness, future implementation and standardization of use of the DT as part of routine care will be recommended.

**Trial registration:**

This study is registered at clinicaltrial.gov march 17, 2010 (NCT01091584).

## Background

With an incidence of more than 1.67 million women yearly, breast cancer is the second most frequently occurring type of all cancers in the world [1]. Despite the reported increase in survival rate, the diagnosis breast cancer has a serious impact on a woman’s life [2, 3]. The National Comprehensive Cancer Network (NCCN) summarizes the problems that patients with cancer may encounter with the word“distress” and defines it as ‘*a multifactorial unpleasant emotional experience of a psychological (cognitive, behavioral, emotional), social and/or spiritual nature that may interfere with the ability to cope effectively with cancer, its physical symptoms and its treatment. Distress extends along a continuum ranging from common normal feelings of vulnerability, sadness and fears to problems that can become disabling such as depression, anxiety, panic, social isolation and existential and spiritual crisis*’[4]. When patients experience distress it impinges on their quality of life and the time for recovery during and after treatment [5–10]. The current NCCN guideline describes that 20-47 % of patients with newly diagnosed and recurrent cancer experience a significant level of distress [4]. Offering basic psychosocial care is a core task for physicians and nurses. Psychosocial care could consist of education about the disease and treatment process, emotional support, as well as support in choosing treatment modalities. For optimal support, it is important to screen for levels of distress and the unmet needs of the patient [11, 12].

### Screening for distress

The Distress Thermometer (DT) has become a worldwide standard screening tool for distress in cancer patients [4, 9, 10, 13–20] that facilitates a systematic approach to distress detection. It consists of a VAS score and a problem list. Without a systematic distress assessment patients are at risk of under diagnosis and treatment [4]. Its use can assist in the timely detection of distress and facilitate early intervention. A screening instrument like the DT provides guidance for discussions with patients. Its use gives attention and focus to psychosocial issues, an increased awareness of distress and more effective communication between healthcare professionals and patients [4].

Recently, an increasing number of papers have been published on the validity of the DT in different languages and on different cut-off points [4, 14–18, 21]. Additionally, the DT is used in studies to measure distress related to various tumor types [19, 20] and at different time points during treatment and follow-up [9, 10, 22, 23]. Studies about the effectiveness of the utilization of the DT are scarce. Based on current knowledge, only one study described a non-blinded randomized controlled trial about the DT in comparison to standard care [24]. In this study the DT was assessed once at baseline and patients filled out questionnaires at 1, 6 and 12 months of follow up. No effect on costs and no significant improvement on the mood states among patients were found [24]. Due to this lack of evidence on effectiveness there is an ongoing discussion about the use of the DT [25–27].

Internationally, it is recommended to implement guidelines to address psychosocial care, with for example the DT, to manage the psychosocial impact of cancer as part of daily oncology care [4, 28, 29].

However, it is still unconfirmed that systematic screening with the DT and a subsequent discussion of the results will lead to improved patients’ quality of life. It is striking that the use of the DT is implemented as standard care worldwide without any evidence of effectiveness. We therefore decided to investigate the added value of using the DT systematic to improve their quality of life by a nurse in oncology care in a randomized controlled trial, in patients diagnosed with breast cancer.

The decision to focus on patients with breast cancer was made for the following reasons; (1) breast cancer has a high incidence, (2) most patients undergo a long treatment process and (3) patients have high survival rates. The high survival rate is essential to be able to measure the effects of the intervention in preventing long-term psychosocial problems.

### Objective

The primary objective of this randomized controlled trial is to evaluate the effect of a nurse-led DT intervention on improving the quality of life of patients with breast cancer who are treated with curative intent, compared to usual care, after approximately two years of follow-up.

## Methods

This study will be reported in accordance with the SPIRIT guidelines [30].

## Study design

**Fig. 1:**
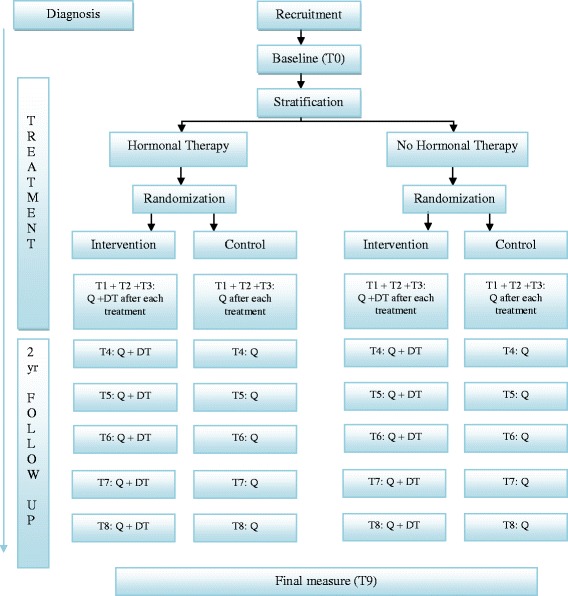
Flow-chart of the nurse intervention project. Q: Questionnaires, DT: Distress Thermometer Intervention includes a thorough assessment using the DT and a discussion of the results by a trained oncology nurse. The questionnaires are: EORTC QLQ-C30 = European Organization for Research and Treatment of Cancer, Quality of Life Questionnaire; QLQ-BR23 = Quality of life- Breast Cancer; HADS = Hospital Anxiety and Depression Scale; IES = Impact of Event Scale; ICQ = Illness Cognition Questionnaire; EQ-6D = EuroQol-6D and a diary

The design of the study (also called Nurse Intervention Project) is a non-blinded randomized controlled trial (Fig. 1). In the intervention group, a thorough assessment using the DT and a discussion of the results by a trained oncology nurse will be added to the usual care. Actions based on the outcomes of the DT will be taken as necessary. The control group will receive the usual care without using the DT. By comparing the results of the intervention group with the control group the effect of the intervention can be determined.

### Participants eligibility

Inclusion criteria: women with histology proven malignancy of the breast; who will receive treatment with curative intent, written and oral fluency in the Dutch language and aged ≥ 18 years.

Exclusion criteria: men, women who have been treated previously for a malignancy (except adequately treated cervix carcinoma in situ and basal cell carcinoma of the skin); women with psychiatric problems that impair adherence to this study.

### Recruitment

Patients will be recruited from the population of newly diagnosed breast cancer patients at the Radboud University Medical Center. Women who have been diagnosed with breast cancer and meet the inclusion criteria, will be asked to participate in this study. The patients will be monitored after surgery, during adjuvant treatment and approximately two years during the follow up. Immediately following diagnosis participants will be verbally briefed by a clinical nurse specialist about the study and given an information pack containing a detailed information sheet and letter of invite to participate in the study. This timing is crucial as it is preferable to collect baseline measurement before start of the first treatment modality. Following receipt of the information package the patient has several days to consider participation in the study. If the patient gives consent for further discussion about the study, the investigator will then be in contact with the patient by telephone or during the next hospital visit to discuss further potential participation. In the time frame between the diagnosis and the start of treatment, the patient usually visits the responsible healthcare professional (surgeon, clinical nurse specialist or oncologist). On that day, if appropriate, the patient will be asked to confirm her participation and baseline measurements will be taken in the hospital or at home. There are paper-and-pencil and electronic versions of the assessment available. Electronic completion reduces the risk of missing data because the patient has to answer each questions before sending. A paper-and-pencil version of the questionnaires will be available for those who are not capable of filling it out electronically.

### Randomization

The expectation is that approximately 75 % of the patients will receive hormonal therapy. Since mood swings and fatigue are known side effects of hormonal therapy [31], we will stratify for hormonal treatment. We therefore will use a randomized block design, prepared by an independent statistician. The patients will be randomized in a 1:1 ratio, immediately after assessment of the adjuvant treatment plan, which includes the use of adjuvant hormonal therapy. Random assignment using sequentially numbering will be done by a physician not involved in the study. The result of the randomization will be communicated by e-mail or mail to the patient by the investigator.

### Intervention

The intervention comprises of support by the trained oncology nurse based on the discussion of the DT in accordance to the protocol for assessing the need for psychosocial care for cancer patients [13]. The intervention is combined with the (follow up) visit to the outpatient clinic.

The DT consists of a thermometer ranging from 0 (no distress) to 10 (extreme distress). In addition the tool contains 47 questions (yes / no answers) related to different issues. The issues have been categorized into: practical issues, family / social issues, emotional issues, religious / spiritual issues, physical issues. The DT concludes with the question: “Would you like to talk with a professional about your problems?” (yes/no/maybe). The cut-off point is 5 [14].

The following steps will be made for each screening moment with the DT:

1. The patient will receive an e-mail or mail about the appointment with the trained oncology nurse, which will take place in combination with regular visits in the outpatient clinic.
2. The patient will fill out out the DT in the outpatient clinic a few minutes before the appointment.
3. The trained oncology nurse will discuss the DT with the patient before or after the visit with the attending healthcare professional. The nurse will ask on which problems the DT score is based and the mentioned problems on the problem list will be discussed. If the patient reports a lot of problems, the nurse will ask the patient to prioritize the problems indicated. At the end, the nurse will ask if the patient would like to be referred to a professional.
4. Time allocated to these meetings will last between 5 – 30 min, depending on the severity of the distress and the nature of the problems.
5. If the patient reports a DT score of <5 the trained oncology nurse will inquire whether the patient is sufficiently in control of her situation. The low distress score and the issues marked on the problem list are discussed briefly. At a score ≥ 5 on the DT, an extensive exploratory conversation between the nurse and the patient will take place. The outcome of this conversation will be discussed in a psychosocial Multi Disciplinary Team (MDT). The MDT has been established to discuss all patients of the intervention group with a score of ≥ 5 on the DT and to discuss patients who personally request additional support. The participants of the MDT are the attending healthcare professional and/or oncologist, the trained oncology nurse, a social worker and clinical psychologists. During the MDT a treatment plan is composed when needed. The nurse will propose this plan to the patient by phone.

Three oncology nurses will be trained by a clinical psychologist to perform the intervention over three sessions. A specific manual will be developed during the training sessions and the intervention. In order to apply consistency in the content and the discussion of the DT with the patients, those three nurses will receive the same training. They should be qualified as a nurse and be knowledgeable in the course and treatment of breast cancer. For financial reasons an independent trained study nurse cannot be hired for this study. Because oncology nurses from the wards have a high risk of contamination the intervention group while being in contact with patients from both the intervention and the control group, they are not suited to deliver the intervention themselves. Therefore, we will select three oncology nurses who are not bedside nurses and are involved in other than breast cancer patient groups on the out-patient clinic. In order to build a trustworthy relationship and give continuity to the care whenever possibly the patient will meet the same oncology nurse at every visit. During this study the DT is not implemented in daily care so the oncology nurses of the departments involved in the study will not use it in daily practice.

A short standard report (as incorporated in the manual) will be filled out after each conversation. To prevent contamination with other professionals, the report of the conversation will not be included in the medical record of the patient. The investigator of the study is also one of the trained oncology nurses who will deliver the interventions. In order to minimize the influence of the investigator on the results of the study, an independent database will be created and an independent statistician will analyze the data.

### Usual care

As already mentioned, the DT will not be implemented in daily care for patients with breast cancer during this study period, therefore no professionals taking care of breast cancer patients will use the DT. The usual care consists of routine follow-up visits with the attending healthcare professional (physician or clinical nurse specialist) according to the Dutch breast cancer guideline [32] (see also measuring time-points). Depending on the judgment of the responsible health care professional, the patients may be referred to other health care professionals, if indicated. No psychosocial MDT is available in usual care.

### Measuring time-points

The baseline measurement will take place preferably before breast surgery or start of the neo-adjuvant chemotherapy. During the first year of treatment the assessments will take place at the end of each treatment modality. In the second and third year, the follow-up visits will be in line with the recommendations of the Dutch Breast Cancer guideline [32]. This means that data will be collected approximately every 3 months during the first and every 6 months in the second year after the completion of adjuvant treatment (except for trastuzumab or hormonal therapy). This will result in a total of 8 – 10 measurements, depending on the number of adjuvant therapies (see Fig. 1). In order to monitor the effects of treatment on the patients’ well-being in both the short- and long-term, patients will be followed for two years after completion of the primary (adjuvant) treatment.

At all measuring time-points both groups will receive questionnaires. The DT is included for both groups at baseline and at the end of the study. At those time-points the results will not be discussed with the patients. Approximately two days before the regular visit to the attending healthcare professional, the patient will be asked to fill out the questionnaire electronically (Radquest software, department of Medical Psychology) or on paper in the hospital or at home. It takes 10–30 min to fill out the questionnaires. Additionally, a diary will be provided to the patient in which the consumed care and work absence has to be noted and recorded. At the moment the patient hands in the diary, a new one is provided by mail or personally. In case of non-response, a reminder will be send within 2 weeks by e-mail or mail.

### Study outcome measures

*Demographic* data and the *use of psychosocial care* are measured with general questionnaires. *Medical disease-specific* data will be collected from the electronic medical record. A checklist will be used to collect the relevant medical records from the patient’s status.

#### Primary outcome measure

The primary outcome will be the global *quality of life* subscale as defined by the European Organization for Research and Treatment of Cancer, Quality of Life Questionnaire- C30 (EORTC QLQ C-30) [33]. The items of the global health and global quality of life scale use a 7-point linear analogue scale (very poor to excellent) [34].

#### Secondary outcomes

The secondary outcomes will be: breast cancer related quality of life, anxiety, depression, emotional distress, coping, illness cognitions and distress (Table 1).

**Table 1:** Measurements and time points of the nurse intervention project

Questionnaires	Target	T0	T1-T8	T9	Response format
Demographic & medical characteristics	Descriptive of the population	X	X	X	Multiple answers
EORTC QLQ-C30	Functional scales: physical, role, emotional, social, and cognitive functioning (15 items)	X	X	X	4 point Likert scale Range 15–60
	Symptom scales: fatigue, pain, nausea/vomiting (7 items)	X	X	X	Range 7–28
	Single symptom items (6 items)	X	X	X	Range 6–24
	Global Health and global quality of life* (2 items)	X	X	X	7 point linear analogue scale Range 2–14
EORTC QLQ- BR23	Functional scales: body image, sexual functioning, sexual enjoyment, future perspective (8 items)	X	X	X	4 point Likert scale Range 8–32
	Symptoms scales: arm symptoms, breast symptoms, systemic therapy side effects, upset by hair loss (15 items)	X	X	X	Range 15-60
DT	General distress	X	X^1^	X	11 point visual analogue Scale: range 0–10
	Problem list (47 items)	X	X^1^	X	Yes/no
	Question: wish for referral (1 item)	X	X^1^	X	Yes/no/maybe
HADS	Emotional distress (14 items) Subscales: Anxiety (7 items) Depression (7 items)	X	X	X	4 point Likert scale Range 0–42 (total scale) Range 0–21 Range 0-21
IES	Coping with the cancer (15 items) Subscales: Intrusion (7 items) Avoidance (8 items)	X	X	X	4 point scale Range 0–75 (total score) Range 0–35 Range 0-40
ICQ	Illness perceptions (18 items) Subscales: Helplessness (6 items) Acceptance (6 items) Perceived benefits (6 items)	X	X	X	4 point Likert scale Range 6–24 Range 6–24 Range 6-24
Diary	Health care use and work absence		X	X	Yes/no, frequency and reason
EQ-6D	Quality of life in relation to economic evaluations (1 item)	X	X	X	Visual Analogue Scale: range 0–100
	Dimensions: mobility, self-care, usual activities, pain/discomfort, anxiety/depression and cognition (6 items)	X	X	X	Range 1–3 for each dimension.

*primary outcome

^1^only in the intervention group

*Abbreviations*: *T0* baseline measurement, *T1-T3* measurement after each treatment and T4-T8 follow up, *T9* final measure, *EORTC QLQ-C30* European organization for research and treatment of cancer, quality of life questionnaire, *QLQ-BR23* quality of life- breast cancer, *DT* distress thermometer, *HADS* hospital anxiety and depression scale, *IES* impact of event scale, *ICQ* illness cognition questionnaire, *EQ-6D* euroQol-6D

Functional and symptom scales of the EORTC QLQ C30 will be used to assess the other dimensions of quality of life (see Table 1). Higher scores on the global and function scales implies good quality of life. On the symptom scales, low scores indicates less intense symptoms hence higher quality of life [33-35].

*Breast cancer related quality of life* will be assessed with the breast cancer related questionnaire EORTC- BR23 which consists of 23 questions and complements the C30 [36].

*Anxiety, depression* and *emotional distress* will be measured with the Hospital Anxiety and Depression Scale (HADS). The HADS has two subscales (anxiety and depression) and a total score of emotional distress. The questionnaire consists of 14 questions with scores ranging from 0 (not at all) to 3 (very much) [37–39].

The presence of *coping problems* will be measured with Impact of Event Scale (IES). This questionnaire provides an inventory of the effects of a shocking event and focuses on the person’s feelings and thoughts over the previous seven days. The IES has two subscales: intrusion and avoidance. The scores range from ‘not at all’, ‘rarely’, ‘sometimes’ and ‘often’[40].

To identify the role of *illness cognitions* in relation to the treatment effectiveness we will use the Illness Cognition Questionnaire (ICQ). There are three subscales: helplessness, acceptance and perceived benefits. The scores range from 1 (none) to 4 (entirely) [41].

*Distress* will also be measured with the DT at baseline and final measurement for both groups (see Fig. 1) [14].

In case we will find differences between both groups on quality of life we will further explore the health care utilization. These data will be gathered with a diary that the patients will take home between measurement time-points. Patients will register their health care utilization, cancer-related absence from work, specific medication and care. Quality of Life in relation to economic evaluations will be measured using the EuroQol-6D (EQ-6D). The EQ-6D comprises both the EQ-5D and an additional dimension namely, cognition. The EQ-5D measures health on five dimensions (mobility, self-care, usual activities, pain/discomfort, anxiety/depression). Every dimension is differentiated in three levels: no problems, some problems, and extreme problems. The EuroQol Visual Analogue Scale (EQ-VAS) will also be used. The EQ-VAS provides a subjective assessment of quality of life on a scale ranging from 0 (worst health) to 100 (best health) [42].

#### Evaluation

At the end of the study, patients will be asked to evaluate their experiences during the study period. In addition, the intervention group will be asked to evaluate their experience of the intervention. The control group will be asked about their need for more psychosocial support during treatment or follow up.

### Data management

We expect most patients will fill out the questionnaires electronically. The results of the questionnaires will be converted to SPSS by a data manager. Only the investigators have access to the coding, storage of all questionnaires and the final dataset. The paper and pencil questionnaires and medical characteristics will be entered in the SPSS database by a research assistant. For the validity of these data, for 10 % of the data double entry of data will be done. A statistician will check data value ranges. All source data will be stored for 15 years.

### Power calculations

Based on prior clinical studies [34] a difference of 10 points in the EORTC QLQ-C30 and its subscales is considered a clinically relevant difference for patients with cancer. The power of the study to detect an effect of 10 points or more is calculated as follows.

The primary outcome is the global quality of life subscale of the EORTC QLQ-C30 (sd = 22.7) at the end of the study [43]. However, we aim for sufficient power for the most important secondary outcomes – the subscales: role function, emotional function, cognitive function and social function of the EORTC QLQ-C30 (clinically relevant difference 10 and sd = 18.7 – 22.8) [43]. Therefore, we are aiming for 84 patients per group. The power of the primary outcome then becomes more than 96 %, and for the relevant subscales at least 80 %, when analyzing these outcomes with adjustment for baseline (i.e., an ANCOVA which has as much power as the *t*-test or more depending on the correlation of the baseline measurement with the measurement at the end of the trial). Taking a drop-out of at most 15 % into account, a total number of 193 patients needs to be included to have sufficient power for the primary and secondary outcomes.

### Statistical analysis

The primary analysis is the comparison of the primary outcome global quality of life subscale of the EORTC QLQ-C30 as measured at the end of the research period analyzed by ANCOVA, i.e., an adjustment for baseline will be included. The secondary analysis of the primary outcome is a repeated measurements analysis of the sequence of the repeated measurements in order to compare the trends between the two groups (mixed model for repeated measurements). Similar analysis will be carried out on the secondary outcomes. Subgroup analysis will be performed on demographic and treatment characteristics.

Missing data will be analyzed with the last observation carried forward method for the ANCOVA and a sensitivity analysis assuming missing data to be missing-at-random will be performed using a mixed model for repeated measurements. Patients who have died, had recurrence or metastasis of the breast cancer, or were diagnosed with another malignancy during the study will be considered to have dropped out of the study from that event onwards.

## Discussion

This study will evaluate the effect of an oncology nurse-led DT intervention compared to usual care on improving the quality of life of patients who are treated for breast cancer with curative intent. The results will contribute to the actual knowledge and the current discussion about using the DT in daily oncology practice [25-27], as most previous studies were performed for validation of the DT. Even though Hollingworth et al. performed an RCT to measure the efficacy of the DT, they used the DT once [24]. In our study, we will offer a nurse-led DT intervention repeatedly in a period of more than two years, which makes our study unique and complementary to the existing literature. Despite a natural recovery of quality of life over time, we will expect additional improvement when using the DT systematically during a longer period. As a primary endpoint, we will use the EORTC QLQ-C30 global quality of life scale. In order to compensate for probable response shift, we will also inspect the secondary outcomes to measure distress reduction. Additionally, the results of this study will give us insight into the trajectories of distress and quality of life from diagnosis to 2-year follow up in the usual care group. The short- and long-term problems of patients with breast cancer will become apparent. Therefore, the outcome of our study may have impact on the future implementation of the DT both nationally and internationally. The strengths of our study are: (1) we will follow the guideline about distress management, (2) we will systematically assess and discuss the DT for approximately 2 years follow up, (3) we will discuss the DT results of all patients in the intervention group with high distress in a psychosocial MDT.

## Conclusion

In conclusion, the aim of our study is to determine the effectiveness of the systematic use of the DT and the subsequent discussion of the results with a trained oncology nurse compared with usual care on the quality of life of the patient with breast cancer. It is anticipated that the results of the study will have impact on the future implementation and standardization of the use of the DT as part of routine care. It is expected that the data collection will be completed early 2016.
